# Health-related quality of life questionnaires in lung cancer trials: a systematic literature review

**DOI:** 10.1186/2191-1991-3-15

**Published:** 2013-05-16

**Authors:** Kathrin Damm, Nicole Roeske, Christian Jacob

**Affiliations:** 1 Center for Health Economics Research Hannover (CHERH), Leibniz University Hannover, Hannover, Germany; 2 Herescon GmbH, Hannover, Germany

**Keywords:** Lung cancer, Health-related quality of life, Questionnaires, Health economics, Utility measurement

## Abstract

**Background:**

Lung cancer is one of the leading causes of cancer deaths. Treatment goals are the relief of symptoms and the increase of overall survival. With the rising number of treatment alternatives, the need for comparable assessments of health-related quality of life (HRQoL) parameters grows. The aim of this paper was to identify and describe measurement instruments applied in lung cancer patients under drug therapy.

**Methods:**

We conducted a systematic literature review at the beginning of 2011 using the electronic database Pubmed.

**Results:**

A total of 43 studies were included in the review. About 17 different measurement instruments were identified, including 5 generic, 5 cancer-specific, 4 lung cancer-specific and 3 symptom-specific questionnaires. In 29 studies at least 2 instruments were used. In most cases these were cancer and lung cancer-specific ones. The most frequently used instruments are the EORTC QLQ-C30 and its lung cancer modules LC13 or LC17. Only 5 studies combined (lung) cancer-specific questionnaires with generic instruments.

**Conclusions:**

The EORTC-C30 and EORTC-LC13 are the most frequently used health-related quality of life measurement instruments in pharmacological lung cancer trials.

## Review

### Introduction

Lung cancer is one of the most frequent cancers in Germany. With more than 47,000 new cases in 2006, lung cancer ranks third among malignant tumors; with a 5-year survival rate of about 15% [1]. Symptoms include cough, coughing up blood, shortness of breath, chronic lung inflammation, chest pain, weakness or loss of appetite. Due to a long term symptom-free course of the disease and non-specific complaints at first, lung cancer in contrast to other tumours is often diagnosed at an advanced stage. Therefore, treatment goals for these patients are symptom relief and an increased overall survival [2]. At the same time therapies that improve survival time are often accompanied by burdensome (toxic) side effects.

Because of the increasing number of therapy lines and treatment alternatives, the declining differences in clinical effectiveness and cost of drugs, the importance of consistent and comparable health-related quality of life (HRQoL) parameters grows - both for medical and health economic evaluation. Up to today, their inclusion in clinical lung cancer trials is generally neglected [2-4].

The questionnaire-based measurement of HRQoL has become standard. To assess the HRQoL in patients with lung cancer, about 50 different instruments are available that directly address to the patient or apply to the practitioner [4]. In general, criteria used to distinguish the various instruments are the aggregated or disaggregated scores, ordinal measures or cardinal scales and the disease specificity (see Table 1) [5].

**Table 1:** Classification of HRQoL-questionnaires

**Classification of HRQoL-questionnaires**					
**Aggregation of results**		**Disease specificity**		**Scaling**	
Separate measurement of different dimensions of HRQoL	Aggregation to an index	Comparison within a group of patients	Comparison between patient groups	Determining a rank order	Determining relative distances
Particularly suitable for medical purposes	Particularly suitable for economic purposes	Particularly suitable for medical purposes	Particularly suitable for economic purposes	Particularly suitable for medical purposes	Particularly suitable for economic purposes

Source: Based on Schöffski O (2007) [5].

In addition to former research by Liu et al. [3], who reviewed and summarized HRQoL measures in kidney cancer, hepatocellular carcinoma, and leukemia, the aim of this systematic literature review is to investigate which questionnaires are applied in lung cancer patients treated with drugs. Using this approach, we examine whether the variety of possible lung cancer measurement instruments is also reflected by research practice.

### Methods

**Figure 1:**
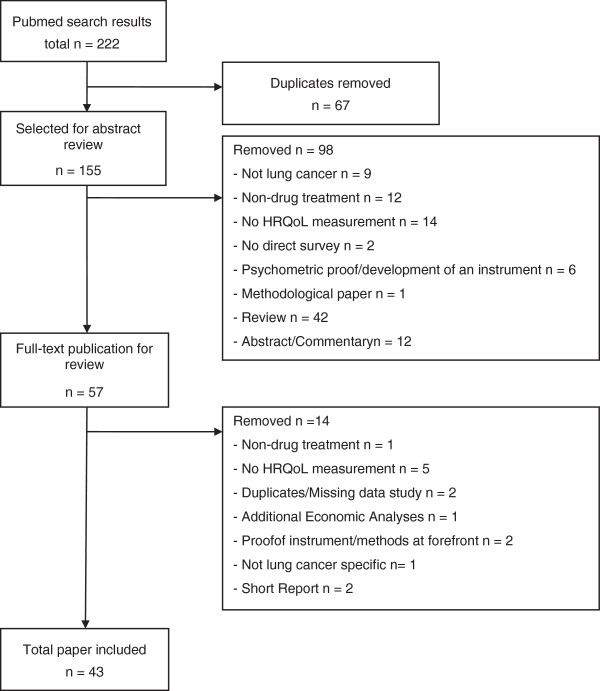
Flow chart of study selection.

Research and documentation were carried out in accordance with the guideline PRISMA (Preferred Reporting Items for Systematic Reviews and Meta-Analyses) [6]. We searched the database PubMed combining the following search terms: “Biological Therapy” (MeSH), “Chemicals and Drugs Category” (MeSH), “Drug Therapy” (MeSH), “Individualized Medicine” (MeSH), “Lung Neoplasms” (MeSH), “Outcome and Process Assessment (Health Care)” (MeSH Major Topic), “Quality of Life” (MeSH Major Topic), “Symptom Palliation” (Free text search, Major Topic). The quality of life associated key words were defined as major topic, to exclude articles that deal with the issue only as a secondary aspect. In addition, a manual search was carried out. In order to focus on current publications, the present review includes literature published in English and German language between 2001 and 2011. Titles, abstracts and full-texts of the identified studies were reviewed independently by three researchers. Exclusion criteria are documented in Figure 1. The identified studies were analyzed concerning the HRQoL results and used questionnaires.

### Results

A total of 43 studies on the HRQoL measurement in lung cancer patients treated with drugs were identified (see Figure 1). The language restriction led only to small deviations from the overall number of identified studies.

Most of the identified literature presents results of phase III clinical trials (see Table 2). In 27 studies HRQoL is a primary endpoint. The majority of studies (n=38) includes patients with non-small cell lung cancer (NSCLC), mainly in stages III/IV. Two studies include patients with small cell lung cancer (SCLC) in all stages. In three studies both NSCLC and SCLC patients entered. Mainly platinum-based drug combinations are trialed. Eight studies investigate EGFR inhibitors: 6 studies examine the effect of gefitinib, 2 the effect of erlotinib.

**Table 2:** Identified literature overview

**Author (Year)**	**Study, Phase tumor type stadium**	**Treatment outline**	**HRQoL endpoints**	**QoL outcome**	**QoL instruments**
**Agteresch HJ et al.** (2002) [7]	Clinical trial NSCLC IIIB/IV	Adenosine 5'-Triphosphate (ATP) vs. non	secondary	No change in appetite and body comparison in the ATP group. Control group lost weight and appetite.	Rotterdam Symptom Checklist (RSCL)
**Baka S et al.** (2005) [8]	Clinical trial, II NSCLC IIIB/IV	Comparison of two different treatment schedules for Gemcitabine	primary	Significant improvement in performance from baseline, no sign. difference between treatment schedules.	Subset Scale from the EORTC QLQ-C30 and LC13, Karnofsky performance scale
**Belani C et al.** (2006) [9]	Clinical trial, III NSCLC IIIB/IV	Comparison of two Docetaxel-platinum regimens with Vinorelbine/Cisplatin	primary	Patients treated with Docetaxel-containing regimen had better QoL and relieved symptoms.	LCSS, EQ-5D
**Bezjak A et al.** (2008) [10]	Clinical trial, III NSCLC IB/II	Adjuvant chemotherapy (Cisplatin and Vinorelbine vs. non)	secondary	After chemotherapy QoL returned to baseline by 9 months compared to 3 months in the control group.	EORTC QLQ-C30
**Bezjak A et al.** (2006) [11]	Clinical trial, III NSCLC advanced	Erlotinib vs. placebo after prior chemotherapy	secondary	Sign. Improvement in physical and global QoL, dypnea, cough, pain, emotional functioning, deterioration in sore mouth and hairloss compared to baseline.	EORTC QLQ-C30,-LC13
**Bianco V et al.** (2001) [12]	Clinical trial NSCLC IIIB/IV	Gemcitabine as single agent therapy in advanced NSCLC in elderly patients (>65)	primary	Improvement in QoL, reduction of symptoms.	Spitzer-Index, Instrumental Activities of Daily Living Scale (IADL), EORTC QLQ-C30,-LC13
**Booton R et al.** (2006) [13]	Clinical trial, III NSCLC III/IV	Docetaxel/Carboplatin vs. Mitomycin C/Cisplatin/Vinblastine vs. Ifosfamide/Cisplatin	secondary	No superiority of one regimen.	Hospital Anxiety and Depression Scale (HADS), EORTC QLQ-C30,-LC13
**Bozcuk H et al.** (2006) [14]	Clinical trial NSCLC IIIB/IV	Examining determinants of a QoL improvement with chemotherapy in patients with advanced NSCLC.	primary	Age, baseline QoL and chemotherapy administration influence the degree of change in QoL.	EORTC QLQ-C30
**Brown J et al.** (2005) [15]	Clinical trial NSCLC IV	Supportive care with vs. without additional chemotherapy	primary	No sign. difference in QoL.	EORTC QLQ-C30,-LC17
**Cella D et al.** (2005) [16]	Clinical trial, II NSCLC advanced	Comparison of two different treatment dosages Gefitinib in heavily pretreated patients	primary and secondary	QoL improvements were correlated with tumor response.	FACT-L
**Chen M.-L.** (2008) [17]	Clinical trial NSCLC IIIB/IV, SCLC	Examining the impact of sleep disturbance of lung cancer patients undergoing chemotherapy on their QoL	primary	Sign. impact on the cognitive function and functional status.	HADS, Pittsburgh Sleep Quality Index (PSQI), Brief Pain Inventory (BPI), EORTC QLQ-C30,-LC13
**Dancey J et al.** (2004) [18]	Clinical trial, III NSCLC IIIB/IV	Docetaxel second-line vs. Best Supportive Care (BSC)	secondary	A trend towards less deterioration in QoL compared to BSC	LCSS EORTC QLQ-C30,-LC13
**de Marinis F et al.** (2008) [19]	Clinical trial, III NSCLC III/IV	Pemetrexed vs. Docetaxel	secondary	Positive response on chemotherapy correlates with symptom improvement.	LCSS
**Gelibter A et al.** (2005) [20]	“Compassionate-Use” NSCLC III/IV	Gefitinib	primary	Improvements in fatigue, insomnia, and pain, deterioration in sore mouth, chest-pain, diarrhea.	EORTC QLQ-C30,-LC13
**Gridelli C et al.** (2003) [21]	Clinical trial, III NSCLC IIIB/IV	Gemcitabine/Vinorelbine vs. Gemcitabine/Cisplatin vs. Vinorelbine/Cisplatin	primary	Global QoL is not improved with Gemcitabine/Vinorelbine.	EORTC QLQ-C30,-LC13
**Grønberg BH et al.** (2009) [22]	Clinical trial, III NSCLC IIIB/IV	First-line Pemetrexed/Carboplatin vs. Gemcitabine/Carboplatin	primary	Pemetrexed/Carboplatin provides similar HRQoL with less need for supportive care.	EORTC QLQ-C30,-LC13
**Helbekkmo N et al.** (2009) [23]	Clinical trial, III NSCLC IIIB/IV	Carboplatin Chatelut AUC/Vinorelbine or Carboplatin/Gemcitabine in patients with a performance status (PS) 2 compared to patients with PS 0/1	primary	PS 2 patients had a more profound improvement of global HRQoL.	EORTC QLQ-C30,-LC13
**Hensing TA et al.** (2003) [24]	Clinical trial, III NSCLC IIIB/IV	4 cycles of Carboplatin/Paclitaxel or until disease progression in patients younger than age 70 compared to those aged 70 years and older	primary	No difference in QoL outcomes.	FACT-L
**LeCaer H et al.** (2005) [25]	Clinical trial, II NSCLC IIIB/IV	Docetaxel monotherapy in elderly patients	primary	QoL remained stable during treatment.	Spitzer-Index EORTC QLQ-C30,-LC13
**Leighl NB et al.** (2005) [26]	Clinical trial, III NSCLC IIIB/IV	Paclitaxel/Carboplatin with or without BMS-275291	secondary	No detailed results presented.	EORTC QLQ-C30,-LC13
**Lilenbaum R et al.** (2007) [27]	Clinical trial, II NSCLC IIIB/IV	Erlotinib vs. Paclitaxel/Carboplatin in patients with a performance status (PS) of 2	secondary	No sign. differences.	EORTC QLQ-LC13
**Maione P et al.** (2005) [28]	Clinical trial, III NSCLC IIIB/IV	The prediction of pretreated QoL on the survival of elderly NSCLC patients treated with chemotherapy	primary	Pretreatments global QoL has a sign. prognostic value for survival of elderly patients with advanced NSCLC treated with chemotherapy.	EORTC QLQ-C30,-LC13
**McQuellon R P et al.** (2002) [29]	Clinical trial, III NSCLC, SCLC	Megestrol Acetate vs. placebo in patients undergoing radiation therapy	primary	No sign. difference in overall QoL.	FACT-General, FACT-L
**Mohan A et al.** (2008) [30]	QoL-study NSCLC III/IV	Cisplatin/Etoposide	primary	Sign. improvement in cough, shortness of breath, pain but not in QoL.	WHOQOL-BREF, Hindi
**Moinpour CM et al.** (2002) [31]	Clinical trial, III NSCLC IIIB/IV	Cisplatin/Vinorelbine vs. Carboplatin/Paclitaxel	secondary	No sign. difference in QoL.	FACT-L
**Morita S et al.** (2003) [32]	Clinical trial, III NSCLC IIIB/IV	Cisplatin/Irinotecan vs. Cisplatin/Vindesine vs. Irinotecan	secondary	Clinical parameters have a sign. effect on QoL in patients undergoing chemotherapy.	QoL questionnaire for cancer patients treated with anti-cancer drugs (QOL-ACD)
**Movsas B et al.** (2005) [33]	Clinical trial NSCLC II/IIIA/B	Paclitaxel/Carboplatin with or without Amifostine	secondary	QoL was not sign. different between the arms.	EORTC QLQ-C30,-LC13
**Mu XL et al.** (2004) [34]	“Compassionate-Use” NSCLC III/IV	Gefitinib	primary	Symptom relief and improvement in QoL.	EORTC QLQ-C30,-LC13
**Natale RB** (2004) [35]	Clinical trial, II NSCLC advanced	Different treatment dosages Gefitinib	secondary	Improvements in symptoms and QoL.	FACT-L
**O’Brian MER et al.** (2006) [36]	Clinical trialSCLC	Best Supportive Care with or without Topotecan in patients with relapsed SCLC	secondary	Slower QoL deterioration and greater symptom control.	Patient self assessment similar to the LCSS, EQ-5D
**Paccagnella A et al.** (2004) [37]	Clinical trial, III NSCLC IIIB/IV	Mitomycin/Vinblastine/Cisplatin vs. Mitomycin/Vinblastine/Carboplatin	primary	Spitzer’s questionnaire showed an improved QoL index for Carboplatin.	Spitzer-Index, EORTC QLQ-C30,-LC13
**Pijls-Johannesma M et al.** (2009) [38]	QoL-study NSCLC I – III, SCLC	Radiotherapy with or without chemotherapy	primary	Overall QoL increases back to baseline within 3 months.	EORTC QLQ-C30,-LC13
**Reck M et al.** (2006) [39]	Clinical trial, III SCLC	Paclitaxel/Carboplatin/Etoposide phosphate vs. Carboplatin/Etoposide phosphate/Vincristine	primary	Paclitaxel-containing regimen sign. improved QoL parameters like global overall QoL.	EORTC QLQ-C30
**Sarna L et al.** (2008) [40]	Clinical trial, III NSCLC II/III	Paclitaxel/Carboplatin with or without Amifostin	primary	QoL was not sign. different between the arms.	EORTC QLQ-C30,-LC13
**Schumacher A et al.** (2003) [41]	Clinical trial, III NSCLC III	Cisplatin/Etoposide followed by either surgery before radiotherapy or radio-chemotherapy before surgery	primary	On QoL no sign. effect was found in or between the two treatments.	EORTC QLQ-C30,-LC13
**Sekine I et al.** (2009) [42]	Clinical trial, III NSCLC IIIB/IV	Gefitinib vs. Docetaxel	secondary	Gefitinib improved aspects of QoL over Docetaxel.	FACT-L
**Sirisinha T et al.** (2005) [43]	N/A NSCLC II-IV	Docetaxel after failure with platinum-based chemotherapy	secondary	No negative impact on overall QoL.	FACT-L
**Thatcher N et al.** (2005) [44]	Clinical trial SCLC	Ifosfamide/Carboplatin/Etoposid/Vincristine vs. standard chemotherapy	primary	No sign. differences regarding QoL.	Rotterdam Symptom Checklist, HADS, EORTC QLQ-C30,-LC13
**Tian JH et al.** (2010) [45]	Clinical trial NSCLC IIIB/IV	Chemotherapy vs. “Feiji Recipe” vs. Chemotherapy/“Feiji Recipe”	primary	"Feiji Recipe" alone or in combination might partially improve QoL.	EORTC QLQ-C30
**Vilmar A et al.** (2010) [46]	Clinical trial, III NSCLC III/IV	Chemotherapy; Determination of biomarker ERCC1	primary	QoL deteriorated sign. among survival-favourable ERCC1-neg. patients	EORTC QLQ-C30,-LC13
**von Plessen C et al.** (2006) [47]	Clinical trial NSCLC IIIB/IV	Optimal duration of palliative Carboplatin with Vinorelbine treatment	primary	No sign. differences between the arms.	EORTC QLQ-C30,-LC13
**Wu WY et al.** (2006) [48]	QoL-study NSCLC IIIB/IV	Gemcitabine/Cisplatin with or without Shenfu Injektion	primary	Shengfu Injektion could improve QoL in patients with Gemcitabine/Cisplatin treatment.	Functional Living Index-Cancer, EORTC QLQ-C30
**Zhang XT et al.** (2005) [49]	“Compassionate-Use” NSCLC III/IV	Gefitinib	primary	Symptom relief and improvement in QoL.	EORTC QLQ-C30,-LC13

EORTC QLQ -C30 = European Organization for Research and Treatment of Cancer Quality of Life Questionnaire-Core (30 Items); EORTC QLQ-LC13 = EORTC QLQ-Lung Cancer (13 Items); EQ-5D = EuroQol-5 Dimensions; FACT-G = Functional Assessment of Cancer Therapy-General; FACT-L = FACT-Lung; HADS = Hospital Anxiety and Depression Scale; LCSS = Lung Cancer Symptom Scale; NSCLC = Non-Small Cell Lung Cancer; QoL=Quality of Life, SCLC = Small Cell Lung Cancer; sign.= significant; WHOQOL = World Health Organization Quality of Life assessment instrument.

#### Identified health-related quality of life (HRQoL) questionnaires

**Figure 2:**
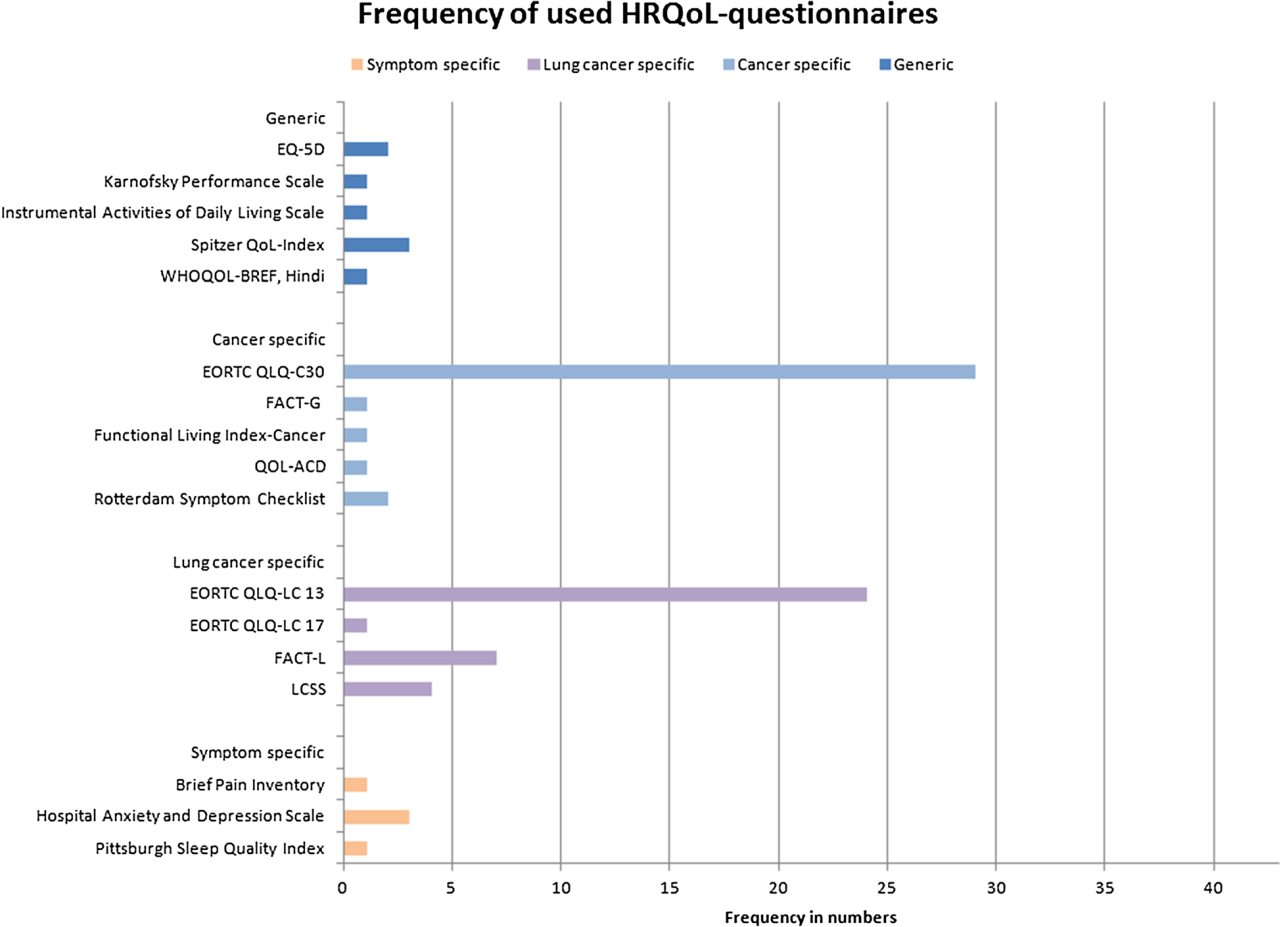
Frequency of used HRQoL-questionnaires.

Overall, 17 different measurement instruments were identified within the included studies (see Figure 2). Five of them are generic, such as the EQ-5D of the EuroQol group or the Spitzer Quality of Life Index. Another 5 instruments are cancer-specific, like the general quality of life questionnaires of the European Organization for Research and Treatment of Cancer (EORTC QLQ-C30) or the FACT-G (Functional Assessment of Cancer Therapy-General) questionnaire. Four instruments are lung cancer-specific, like the lung cancer modules of the EORTC and the FACT-L (Lung) questionnaire as well as the Lung Cancer Symptom Scale (LCSS). The remaining 3 questionnaires are symptom-specific, such as the Hospital Anxiety and Depression Scale (HADS) or the Brief Pain Index (BPI).

The most frequently used instrument is the EORTC QLQ-C30 (*n* = 29), a general questionnaire designed for (self- or interviewer administration) use in cancer patient populations [50]. This 30-item multi-dimensional questionnaire is available in over 60 languages and contains 4 domains (functional and symptom scales, global quality of life, and single items) requested by 4-point Likert or visual analogue scales. Its lung cancer specific module LC13 (13 additional items) is also used widely.

The FACT questionnaires are less frequently used (*n* = 8). Here, the FACT-G (General) contains 27 multi-dimensional items (physical, emotional, social and functional well-being) scored on 4-point scales [51], available in more than 50 languages. The lung cancer questionnaire (FACT-L) is a combination of the FACT-G and disease specific items (in total 37). Further information about the questionnaires as well as comparisons of possible instruments for lung cancer patients can e.g. be found on PROQOLID [52], and in Liu et al.[3], Camps et al.[4] or Damm et al. [53].

In 29 of our identified studies (67%) at least two instruments were used, mostly cancer and lung cancer-specific ones (see Table 2). In 23 studies (53%) the EORTC QLQ-C30 in conjunction with the LC13 module was used. Five studies combined disease-specific (cancer or lung cancer) questionnaires with generic instruments; two times the EQ-5D was applied.

#### Content-related results of the identified literature

Because of the different study populations and treatment regimes it is not possible to compare all the different studies in terms of HRQoL (see Table 2). However we tried to arrange some groups of HRQoL findings.

A majority of the included trials comparing various agent regimes shows no significant differences between treatment arms [13,15,21,29,31,41,44,47]. Another group of studies report cautious assumptions of HRQoL improvements [18,22,37]. Solely, Belani et al. and Reck et al. could show HRQoL-regarded superiority for paclitaxel or docetaxel containing regimes compared to vincristine or vinorelbine/cisplatin [9,39].

With regard to the EGFR inhibitors Gelibter et al., Mu et al., and Zhang et al. all demonstrated symptom relief and improvement in HRQoL by the compassionate use of gefitinib in highly advanced NSCLC patients [20,34,49]. Cella et al. and Natale et al. reported on HRQoL improvements after the administration of gefitinib, compared to baseline in heavily pretreated patients and the correlation of these improvements to the tumour response [16,35].

In terms of erlotinib, Lilenbaum et al. could not show significant improvements in progression-free survival, median survival, and HRQoL in comparison to standard chemotherapy [27]. Bezjak et al. showed significant improvements in HRQoL, if erlotinib was given as second line treatment after chemotherapy [11].

Three studies investigated the application of cytostatic agents in elderly patients. Bianco et al. showed improvements in HRQoL for gemcitabine as a single agent therapy [12], Hensing et al. demonstrated that the application of carboplatin/paclitaxel has no significantly different impact on HRQoL between younger (<70 years) and elderly patients [24]. LeCaer et al. showed stable HRQoL values during docetaxel monotherapy [25].

Movsas et al. and Sarna et al. reported no significant HRQoL differences between treatment regimes in combination with or without amifostine [33,40].

A last group of studies showed influences on HRQoL values, e.g. age and baseline quality of life (QoL) [Bozcuk et al.], the cognitive function [Chen], as well as clinical parameters Morita et al. [14,17,32].

## Discussion

The present study continues the work of previous reviews like the one of Liu et al. for the indication of lung cancer [3].

HRQoL measurement obtains a twofold meaning in the field of lung cancer medication. This is due to the often severe (toxic) therapeutic side effects, but also because of the high demand for symptom palliation. However, the measurement of HRQoL in respective trials is still not consistent and barely comparable [2-4].

By far the most frequently used questionnaire is the EORTC QLQ-C30 in conjunction with the lung cancer-specific module LC13. In comparison, even other lung cancer specific instruments like the FACT-L and the lung cancer symptom scale (LCSS) are only used in relatively few studies [4]. The same applies to the generic instruments. Especially the EQ-5D, which is relevant for health economic evaluations, is rarely used. However, it turns out that, besides the dominant EORTC instruments, a broad portfolio of other questionnaires is applied in different varieties and combinations. This also includes highly sensitive symptom-specific questionnaires. The comparability of these study results thereby is restricted.

A comparison with further literature shows that our results are e.g. in line with Liu et al. and also with Salvo et al. [3,54]. The latter conducted a literature review, published in 2009, searching for quality of life measurement instruments in cancer patients receiving palliative radiotherapy for symptomatic lung cancer. The authors also concluded that EORTC QLQ-C30 was the most commonly used questionnaire (in 13 of 20 trials). Of those 13 studies, 8 also used the LC13 module. The FACT-L was used in only 2 of the included 20 trials. Salvo et al. furthermore considered that HRQoL was of low priority as an endpoint and that measures created for lung cancer patients were underused.

Montazeri et al. identified the EORTC QLQ-C30 and the EORTC QLQ-LC13 as the most sophisticated questionnaires, compared to 50 other instruments to measure the HRQoL in lung cancer patients [55]. This might be one reason for their common use. Another might be the specific symptoms of the lung cancer disease. Moreover, the disease-specific, ordinal scaled profile instruments allow a separate evaluation of therapeutic effects for various dimensions. However, for the comparing of costs and effectiveness in health economic evaluations, an index value is required.

Many of the identified questionnaires allow for the aggregation into one index value, but this is by simply summarising the dimension values. Thus, it is based on an arbitrary weighting of individual aspects of quality of life. Such a value is met with the criticism of being barely interpretable and informative. In addition, the ordinal scaling of the items is inadequate for the economic evaluation of treatment alternatives. Here it is crucial, whether significant improvements in HRQoL can be achieved in the situation of relatively expensive therapy. The EQ-5D is the only identified questionnaire in this study that measures the quality of life in a cardinal index value and thus meets the requirements of cost-utility analyzes. However, as a generic instrument, the EQ-5D is limited by the disadvantage of a relatively low sensitivity in the measurement of small changes in quality of life. This is probably one reason for its relatively rare use in lung cancer studies.

Nevertheless, there are ways in which the identified questionnaires could be used in economic evaluations. One possibility is to transform the most widely used questionnaires EORTC QLQ-C30 and QLQ-LC13 into preference-based versions, similar to the development of the SF-36. This was already considered by the EORTC in the early 90s [50].

In what follows, we highlight some limitations with respect to our research strategy and the results of literature reviews in general. One major limitation of literature reviews is the publication bias. We only considered published full-text study results, no short reports, no conference presentations or study protocols. A comparison with the database clinicaltrials.gov might be a more complete research approach.

Furthermore the restriction of the publication time period might also be a limitation. However, we wanted to analyse the actual situation. In addition, the developments in the fields of HRQoL questionnaires could lead to problems of interpretation.

Our literature search was performed in the Pubmed database only. This is another limitation of our work. Nevertheless, we tried to limit this bias by conducting an additional manual search.

## Conclusions

The cancer-specific EORTC QLQ-C30 with the lung cancer-specific LC13 module are the dominant instruments in HRQoL measurement in lung cancer studies. Besides these instruments, a broad spectrum of other questionnaires is used in different varieties and combinations. Only a small number of studies used generic instruments like the EQ-5D.

## Competing interests

The authors declare that they have no competing interests.

## Authors’ contributions

KD and NR conducted the literature search. All authors reviewed the studies and wrote the manuscript. All authors read and approved the final manuscript.

## References

[B1] Robert Koch Institut Gesellschaft der epidemiologischen Krebsregister in Deutschland e. V: Krebs in Deutschland 2005/2006 2010 Berlin: Häufigkeiten und Trends

[B2] Reck M Quality of life as an endpoint of clinical trials Lung Cancer 2009 3 Suppl 1 22 2318722686

[B3] Liu J Mittendorf T von der Schulenburg JM A structured review and guide through studies on health-related quality of life in kidney cancer, hepatocellular carcinoma, and leukemia Cancer Invest 2010 3 312 322 10.3109/07357900903287022 19863345

[B4] Camps C del Pozo N Blasco A Blasco P Sirera R Importance of quality of life in patients with Non-small-cell lung cancer Clin Lung Cancer 2009 3 83 90 10.3816/CLC.2009.n.010 19362950

[B5] Schöffski O Schöffski O, Von der Schulenburg JM Lebensqualität als Ergebnisparameter in gesundheitsökonomischen Studien Gesundheitsökonomische Evaluationen 2007 Berlin: Heidelberg: Springer 321 334

[B6] Moher D Liberati A Tetzlaff J Altman DG Preferred reporting items for systematic reviews and meta-analyses: the PRISMA statement BMJ 2009 3 332 336 PMC3090117 21603045

[B7] Agteresch HJ Rietveld T Kerkhofs LG van den Berg JW Wilson JH Dagnelie PC Beneficial effects of adenosine triphosphate on nutritional status in advanced lung cancer patients: a randomized clinical trial J Clin Oncol 2002 3 371 378 10.1200/JCO.20.2.371 11786563

[B8] Baka S Ashcroft L Anderson H Lind M Burt P Stout R Dowd I Smith D Lorigan P Thatcher N Randomized phase II study of Two gemcitabine schedules for patients with impaired performance status (karnofsky performance status 70) and advanced Non-small-cell lung cancer J Clin Oncol 2005 3 2136 2144 10.1200/JCO.2005.01.003 15713598

[B9] Belani CP Pereira JR von Pawel J Pluzanska A Gorbounova V Kaukel E Mattson KV Ramlau R Szczesna A Fidias P Millward M Fossella F TAX 326 study group Effect of chemotherapy for advanced Non-small cell lung cancer on Patients’ quality of life – a randomized controlled trial Lung Cancer 2006 3 231 239 10.1016/j.lungcan.2006.05.003 16787687

[B10] Bezjak A Lee CW Ding K Brundage M Winton T Graham B Whitehead M Johnson DH Livingston RB Seymour L Shepherd FA Quality-of-life outcomes for adjuvant chemotherapy in early-stage Non–small-cell lung cancer: results from a randomized trial, JBR.10 J Clin Oncol 2008 3 5052 5059 10.1200/JCO.2007.12.6094 18809617 PMC2652099

[B11] Bezjak A Tu D Seymour L Clark G Trajkovic A Zukin M Ayoub J Lago S de Albuquerque Ribeiro R Gerogianni A Cyjon A Noble J Laberge F Chan RT Fenton D Von Pawel J Reck M Shepherd FA National Cancer Institute of Canada Clinical Trials Group Study BR.21 Symptom improvement in lung cancer patients treated with erlotinib: quality of life analysis of the national cancer institute of Canada clinical trials group study BR.21 J Clin Oncol 2006 3 3831 3837 10.1200/JCO.2006.05.8073 16921034

[B12] Bianco V Di Girolamo B Pignatelli E Speranza I Florio G Gemma D Girolami M Vietri F Marchei P Gemcitabine as single agent therapy in advanced Non small-cell lung cancer and quality of life in the elderly Panminerva Med 2001 3 15 19 11319513

[B13] Booton R Lorigan P Anderson H Baka S Ashcroft L Nicolson M O’Brien M Dunlop D O'Byrne K Laurence V Snee M Dark G Thatcher N A phase III trial of docetaxel/carboplatin versus mitomycin C/ifosfamide/cisplatin (MIC) or mitomycin C/vinblastine/cisplatin (MVP) in patients with advanced Non-small-cell lung cancer: a randomised multicenter trial of the british thoracic oncology group (BTOG1) Ann Oncol 2006 3 1111 1119 10.1093/annonc/mdl078 16603599

[B14] Bozcuk H Dalmis B Samur M Ozdogan M Artac M Savas B Quality of life in patients with advanced Non–small cell lung cancer Cancer Nurs 2006 3 104 110 10.1097/00002820-200603000-00004 16565619

[B15] Brown J Thorpe H Napp V Fairlamb DJ Gower NH Milroy R Parmar MK Rudd RM Spiro SG Stephens RJ Waller D West P Peake MD Assessment of quality of life in the supportive care setting of the Big lung trial in Non-small-cell lung cancer J Clin Oncol 2005 3 7417 7424 10.1200/JCO.2005.09.158 16157935

[B16] Cella D Herbst RS Lynch TJ Prager D Belani CP Schiller JH Heyes A Ochs JS Wolf MK Kay AC Kris MG Natale RB Clinically meaningful improvement in symptoms and quality of life for patients with Non–small-cell lung cancer receiving gefitinib in a randomized controlled trial J Clin Oncol 2005 3 2946 2954 10.1200/JCO.2005.05.153 15699477

[B17] Chen ML Yu CT Yang CH Sleep disturbances and quality of life in lung cancer patients undergoing chemotherapy Lung Cancer 2008 3 391 400 10.1016/j.lungcan.2008.03.016 18468718

[B18] Dancey J Shepherd FA Gralla RJ Kim YS Quality of life assessment of second-line docetaxel versus best supportive care in patients with Non-small-cell lung cancer previously treated with platinum-based chemotherapy: results of a prospective, randomized phase III trial Lung Cancer 2004 3 183 194 10.1016/j.lungcan.2003.09.001 14739039

[B19] de Marinis F Pereira JR Fossella F Perry MC Reck M Salzberg M Jassem J Peterson P Liepa AM Moore P Gralla RJ Lung cancer symptom scale outcomes in relation to standard efficacy measures – an analysis of the phase III study of pemetrexed versus docetaxel in advanced Non-small cell lung cancer J Thorac Oncol 2008 3 30 36 10.1097/JTO.0b013e31815e8b48 18166838

[B20] Gelibter A Ceribelli A Pollera CF Milella M Moscetti L Sperduti I Cognetti F Impact of gefitinib (‘Iressa’) treatment on the quality of life of patients with advanced non-small-cell lung cancer J Cancer Res Clin Oncol 2005 3 783 788 10.1007/s00432-005-0029-9 16184381 PMC12161201

[B21] Gridelli C Gallo C Shepherd FA Illiano A Piantedosi F Robbiati SF Manzione L Barbera S Frontini L Veltri E Findlay B Cigolari S Myers R Ianniello GP Gebbia V Gasparini G Fava S Hirsh V Bezjak A Seymour L Perrone F Gemcitabine plus vinorelbine compared with cisplatin plus vinorelbine or cisplatin plus gemcitabine for advanced Non-small cell lung cancer: a phase III trial of the Italian GEMVIN investigators and the national cancer institute of Canada clinical trials group J Clin Oncol 2003 3 3025 3034 10.1200/JCO.2003.06.099 12837810

[B22] Grønberg BH Bremnes RM Fløtten O Amundsen T Brunsvig PF Hjelde HH Kaasa S von Plessen C Stornes F Tollåli T Wammer F Aasebø U Sundstrøm S Phase III study by the Norwegian lung cancer study group: pemetrexed plus carboplatin compared with gemcitabine plus carboplatin as first-line chemotherapy in advanced Non–small-cell lung cancer J Clin Oncol 2009 3 3217 3224 10.1200/JCO.2008.20.9114 19433683

[B23] Helbekkmo N Strøm HH Sundstrøm SH Aasebø U Von Plessen C Bremnes RM Norwegian Lung Cancer Study Group Chemotherapy and quality of life in NSCLC PS 2 patients Acta Oncol 2009 3 1019 1025 19274496 10.1080/02841860902795240

[B24] Hensing TA Peterman AH Schell MJ Lee JH Socinski MA The impact of Age on toxicity, response rate, quality of life, and survival in patients with advanced, stage IIIB or IV nonsmall cell lung carcinoma treated with carboplatin and paclitaxel Cancer 2003 3 779 788 10.1002/cncr.11548 12910523

[B25] LeCaer H Delhoume JY Thomas PA Berard H Paillotin D Barriere JR Gimenez C Vergnenegre A Muller P Auquier P Perol M Multicenter phase II trial of carboplatin/vinorelbine in elderly patients with advanced Non–small-cell lung cancer—efficacy and impact on quality of life: groupe français de pneumo-cancérologie study 9902 Clin Lung Cancer 2005 3 114 120 10.3816/CLC.2005.n.026 16179098

[B26] Leighl NB Paz-Ares L Douillard JY Peschel C Arnold A Depierre A Santoro A Betticher DC Gatzemeier U Jassem J Crawford J Tu D Bezjak A Humphrey JS Voi M Galbraith S Hann K Seymour L Shepherd FA Randomized phase III study of matrix metalloproteinase inhibitor BMS-275291 in combination with paclitaxel and carboplatin in advanced Non–small-cell lung cancer: national cancer institute of Canada–clinical trials group study BR.18 J Clin Oncol 2005 3 2831 2839 15837997 10.1200/JCO.2005.04.044

[B27] Lilenbaum R Axelrod R Thomas S Dowlati A Seigel L Albert D Witt K Botkin D Randomized phase II trial of erlotinib or standard chemotherapy in patients with advanced non-small-cell lung cancer and a performance status of 2 J Clin Oncol 2008 3 863 869 10.1200/JCO.2007.13.2720 18281658

[B28] Maione P Perrone F Gallo C Manzione L Piantedosi F Barbera S Cigolari S Rosetti F Piazza E Robbiati SF Bertetto O Novello S Migliorino MR Favaretto A Spatafora M Ferraù F Frontini L Bearz A Repetto L Gridelli C Barletta E Barzelloni ML Iaffaioli RV De Maio E Di Maio M De Feo G Sigoriello G Chiodini P Cioffi A Guardasole V Pretreatment quality of life and functional status assessment significantly predict survival of elderly patients with advanced Non–small-cell lung cancer receiving chemotherapy: a prognostic analysis of the multicenter Italian lung cancer in the elderly study J Clin Oncol 2005 3 6865 6872 10.1200/JCO.2005.02.527 16192578

[B29] McQuellon RP Moose DB Russell GB Case LD Greven K Stevens M Shaw EG Supportive Use of magestrol acetate (megace) with head/neck and lung cancer patients receiving radiation therapy Int J Radiat Oncol Biol Phys 2002 3 1180 1185 10.1016/S0360-3016(01)02782-1 11955728

[B30] Mohan A Singh P Kumar S Mohan C Pathak AK Pandey RM Guleria R Effect of change in symptoms, respiratory status, nutritional profile and quality of life on response to treatment for advanced Non-small cell lung cancer Asian Pac J Cancer Prev 2008 3 557 562 19256738

[B31] Moinpour CM Lyons B Grevstad PK Lovato LC Crowley J Czaplicki K Buckner ZM Ganz PA Kelly K Gandara DR Quality of life in advanced Non-small-cell lung cancer: results of a southwest oncology group randomized trial Qual Life Res 2002 3 115 126 10.1023/A:1015048908822 12018735

[B32] Morita S Kobayashi K Eguchi K Matsumoto T Shibuya M Yamaji Y Sakamoto J Ohashi Y Influence of clinical parameters on quality of life during chemotherapy in patients with advanced Non-small cell lung cancer: application of a general linear model Jpn J Clin Oncol 2003 3 470 476 10.1093/jjco/hyg083 14594941

[B33] Movsas B Scott C Langer C Werner-Wasik M Nicolaou N Komaki R Machtay M Smith C Axelrod R Sarna L Wasserman T Byhardt R Randomized trial of amifostine in locally advanced Non–small-cell lung cancer patients receiving chemotherapy and hyperfractionated radiation: radiation therapy oncology group trial 98-01 J Clin Oncol 2005 3 2145 2154 10.1200/JCO.2005.07.167 15800308

[B34] Mu XL Li LY Zhang XT Wang SL Wang MZ Evaluation of safety and efficacy of gefitinib (‘iressa’, zd1839) as monotherapy in a series of Chinese patients with advanced non-small-cell lung cancer: experience from a compassionate-use programme BMC Cancer 2004 3 1 8 10.1186/1471-2407-4-1 15318946 PMC516034

[B35] Natale RB Effects of ZD1839 (iressa, gefitinib) treatment on symptoms and quality of life in patients with advanced Non-small cell lung cancer Semin Oncol 2004 3 23 30 15206079 10.1053/j.seminoncol.2004.04.010

[B36] O’Brien ME Ciuleanu TE Tsekov H Shparyk Y Cuceviá B Juhasz G Thatcher N Ross GA Dane GC Crofts T Phase III trial comparing supportive care alone with supportive care with oral topotecan in patients with relapsed small-cell lung cancer J Clin Oncol 2006 3 5441 5447 10.1200/JCO.2006.06.5821 17135646

[B37] Paccagnella A Favaretto A Oniga F Barbieri F Ceresoli G Torri W Villa E Verusio C Cetto GL Santo A De Pangher V Artioli F Cacciani GC Parodi G Soresi F Ghi MG Morabito A Biason R Giusto M Mosconi P Chiarion Sileni V GSTVP (Gruppo di Studio Tumori Polmonari del Veneto) Cisplatin versus carboplatin in combination with mitomycin and vinblastine in advanced Non-small cell lung cancer. A multicenter, randomized phase III trial Lung Cancer 2004 3 83 91 10.1016/S0169-5002(03)00280-0 14698542

[B38] Pijls-Johannesma M Houben R Boersma L High-dose radiotherapy or concurrent chemo-radiation in lung cancer patients only induces a temporary, reversible decline in QoL Radiother Oncol 2009 3 443 448 10.1016/j.radonc.2009.02.010 19297049

[B39] Reck M von Pawel J Macha HN Kaukel E Deppermann KM Bonnet R Ulm K Hessler S Gatzemeier U Efficient palliation in patients with small-cell lung cancer by a combination of paclitaxel, etoposide and carboplatin: quality of life and 6-Years’-follow-Up results from a randomised phase III trial Lung Cancer 2006 3 67 75 10.1016/j.lungcan.2006.04.001 16713013

[B40] Sarna L Swann S Langer C Clinically meaningful differences in patient-reported outcomes with amifostine in combination with chemoradiation for locally advanced Non-small cell lung cancer: an analysis of RTOG 9801 Int J Radiat Oncol Biol Phys 2008 3 1378 1384 10.1016/j.ijrobp.2008.03.003 18501528

[B41] Schumacher A Riesenbeck D Braunheim M Wewers D Heinecke A Semik M Hoffknecht P Macha HN Klinke F Schmidt EW Willich N Berdel WE Thomas M German Lung Cancer Cooperative Group Combined modality treatment for locally advanced Non-small cell lung cancer: preoperative chemoradiation does Not result in a poorer quality of life Lung Cancer 2004 3 89 97 10.1016/j.lungcan.2003.10.004 15013587

[B42] Sekine I Ichinose Y Nishiwaki Y Yamamoto N Tsuboi M Nakagawa K Shinkai T Negoro S Imamura F Eguchi K Takeda K Itoh Y Tamura T Saijo N Fukuoka M Quality of life and disease-related symptoms in previously treated Japanese patients with Non-small cell lung cancer: results of a randomized phase III study (V-15-32) of gefitinib versus docetaxel Ann Oncol 2009 3 1483 1488 10.1093/annonc/mdp031 19282468

[B43] Sirisinha T Sirilertrakul S Jirajarus M Ratanatharathorn V Doxetaxel in previously treated Non-small cell lung cancer patients: clinical efficacy and quality of life Southeast Asian J Trop Med Public Health 2005 3 246 253 15906678

[B44] Thatcher N Qian W Clark PI Hopwood P Sambrook RJ Owens R Stephens RJ Girling DJ Ifosfamide, carboplatin, and etoposide with midcycle vincristine versus standard chemotherapy in patients with small-cell lung cancer and good performance status: clinical and quality-of-life results of the British medical research council multicenter randomized LU21 trial J Clin Oncol 2005 3 8371 8379 10.1200/JCO.2004.00.9969 16293867

[B45] Tian JH Liu LS Shi ZM Zhou ZY Wang L A randomized controlled pilot trial of “feiji recipe” on quality of life of Non-small cell lung cancer patients Am J Chin Med 2010 3 15 25 10.1142/S0192415X10007646 20128041

[B46] Vilmar A Santoni-Rugiu E Sørensen JB ERCC1, Toxicity and quality of life in advanced NSCLC patients randomized in a large multicentre phase III trial Eur J Cancer 2010 3 1554 1562 10.1016/j.ejca.2010.02.045 20395129

[B47] Von Plessen C Bergman B Andresen O Bremnes RM Sundstrom S Gilleryd M Stephens R Vilsvik J Aasebo U Sorenson S Palliative chemotherapy beyond three courses conveys No survival or consistent quality-of-life benefits in advanced Non-small cell lung cancer Br J Cancer 2006 3 966 973 10.1038/sj.bjc.6603383 17047644 PMC2360695

[B48] Wu WY Long SQ Zhang HB Chai XS Deng H Xue XG Wang B Luo HY Liu WS Improvement of quality of life with shenfu injection in Non small-cell lung cancer patients treated with gemcitabine plus cisplatin regimen Chin J Integr Med 2006 3 50 54 10.1007/BF02857431 16571285

[B49] Zhang XT Li LY Wang SL Mu XL Wang MZ Song W Improvements in quality of life and disease-related symptoms in patients with advanced Non-small cell lung cancer treated with gefitinib Chin Med J 2005 3 1661 1664 16232354

[B50] Aaronson NK Ahmedzai S Bergman B Bullinger M Cull A Duez NJ Filiberti A Flechtner H Fleishman SB de Haes JC Stein Kaasa S Klee M Osoba D Razavi D Rofe PB Schraub S Sneeuw K Sullivan M Takeda F The European organization for research and treatment of cancer QLQ-C30: a quality-of-life instrument for use in international clinical trials in oncology J Natl Cancer Inst 1993 3 365 376 10.1093/jnci/85.5.365 8433390

[B51] Cella DF Tulsky DS Gray G Sarafian B Linn E Bonomi A Silberman M Yellen SB Winicour P Brannon J The functional assessment of cancer therapy scale: development and validation of the general measure J Clin Oncol 1993 3 570 579 8445433 10.1200/JCO.1993.11.3.570

[B52] PROQOLID - Patient-Reported Outcome and Quality of life Instruments Databasehttp://www.proqolid.org/

[B53] Damm K Jacob C Mittendorf T von der Schulenburg JM **Lebensqualitätsmessung in klinischen Studien beim Lungenkarzinom – Übersicht anhand der Datenbank ClinicalTrials.gov** [Measuring Health Related Quality of Life in Lung Cancer Clinical Trials – An Overview of the Database ClinicalTrials.gov] PharmacoEconomics - German Research Articles 2012 3 3 15 10.1007/BF03320774

[B54] Salvo N Hadi S Napolskikh J Goh P Sinclair E Chow E Quality of life measurement in cancer patients recieving palliative radiotherapy for symptomatic lung cancer: a literature review Current Oncology 2009 3 16 28 19370175 10.3747/co.v16i2.376 PMC2669235

[B55] Montazeri A Gillis CR McEwen J Quality of life in patients with lung cancer: a review of literature from 1970 to 1995 Chest 1998 3 467 481 10.1378/chest.113.2.467 9498968

